# Heterozygous Mutation in Adenosine Deaminase Gene in a Patient With Severe Lymphopenia Following Corticosteroid Treatment of Autoimmune Hemolytic Anemia

**DOI:** 10.3389/fped.2018.00272

**Published:** 2018-10-01

**Authors:** Serena I. Tripodi, Paola Corti, Silvia Giliani, Arnalda Lanfranchi, Andrea Biondi, Raffaele Badolato

**Affiliations:** ^1^Department of Pediatrics, University of Brescia, Spedali Civili Hospital, Brescia, Italy; ^2^Department of Pediatrics, University of Milan-Bicocca, Monza, Italy; ^3^Cytogenetic and Medical Genetics Unit and “A. Nocivelli” Institute for Molecular Medicine, Spedali Civili Hospital and Department of Molecular and Translational Medicine, University of Brescia, Brescia, Italy; ^4^Stem Cell Laboratory, Section of Hematology and Blood Coagulation, Spedali Civili Hospital, Brescia, Italy

**Keywords:** ADA, lymphopenia, corticosteroids, primary immunodeficiency, autoimmune diseases

## Abstract

We describe a previously healthy 14-year-old girl with acute onset autoimmune hemolytic anemia, associated with severe but transient lymphopenia during corticosteroid therapy, without infectious episodes during follow-up. After detailed investigations to rule out an underlying immunodeficiency, we detected a heterozygous *ADA* gene mutation. This was associated with slightly increased blood levels of adenosine and deoxyadenosine nucleotides and with reduced ADA activity in red blood cells, but within the normal range. This observation suggests that heterozygous ADA mutation might be a predisposing factor for lymphopenia in patients receiving corticosteroid therapy.

## Background

Adenosine Deaminase (ADA) deficiency is a rare autosomal recessive disease, due to mutations of *ADA* gene, the purine salvage enzyme, located on chromosome 20q12-q13.11, that catalyzes the deamination of adenosine and 2′-deoxyadenosine (1–3). It is a systemic disorder of purine metabolism, that leads to the accumulation of the toxic metabolites, deoxyadenosine triphosphate and deoxyadenosine. ADA is ubiquitously expressed in all tissues of the body but most profoundly affects lymphocyte development and function, leading to severe combined immunodeficiency (SCID) (4). T-cell disorders primarily result in infections caused by viral and opportunistic organisms, whereas B-lymphocyte deficiency leads to hypogammaglobulinemia and susceptibility to bacterial infections (5). Unlike other forms of SCID, ADA deficiency also results in non-immunologic manifestations: growth failure, sensorineural hearing loss (6), behavioral abnormalities (7), neurological disorders such as spasticity, ataxia, tremors, cognitive, and neurosensory defects (8), non-infectious pulmonary disease (9), hepatic dysfunction (10), and skeletal dysplasia (11).

There is a quantitative relationship between residual ADA activity and the metabolic and clinical phenotype (12). The majority of ADA-deficient patients present under the age of 6 months with chronic diarrhea, failure to thrive, and interstitial pneumonia (13). About 15% of ADA-SCID cases occur later in childhood or beyond (4). Delayed or late-onset patients have significant immunodeficiency, but variable clinical manifestations (14). These forms show progressive immunological and clinical deterioration, sometimes associated with autoimmune manifestations, including autoimmune hypothyroidism, diabetes mellitus, hemolytic anemia, and immune thrombocytopenia (15–17). Although autoimmunity is frequently observed in primary immunodeficiencies, there is accumulating evidence that ADA deficiency predisposes to this phenomenon not only through general mechanisms of immune dysregulation, but also through specific alterations caused by the accumulating ADA metabolites (17).

## Case presentation

A 14-years-old girl came to our attention because of severe and persistent lymphopenia during an episode of autoimmune hemolytic anemia. Her familiar history was negative for invasive infections and autoimmune diseases. Patient medical history was unremarkable for infections. In addition, previous blood counts were normal. The study conformed to all the protocols of Asst Spedali Civili of Brescia. Informed consent for blood tests and genetic studies was obtained from her parents.

She presented with acute onset anemia (hemoglobin 5.5 g/dl) with positive direct antiglobulin test (Coombs test, IgG 2+), normal platelets (299.000/μL), and white cell count (total leucocytes 5.760/μL, neutrophils 4.160/μL, lymphocytes 1.330/μL). At the beginning she was treated with oral prednisone (2 mg/kg/day), but poor response to the treatment was observed. Therefore, she was switched to four intravenous pulses of methylprednisolone each one at 2 mg/kg within 72 h, followed by intravenous immunoglobulins (1 g/kg).

**Figure 1:**
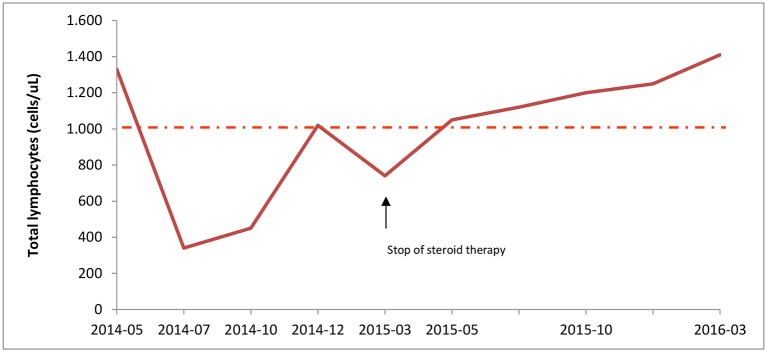
Lymphocyte counts during corticosteroid therapy. Total lymphocytes count was measured at diagnosis (05/2014) until the end of patient follow-up. Steroid treatment, started on May 2014, led to severe lymphopenia; but, immunosuppressive therapy withdrawal on March 2015 resulted in normal lymphocyte count. The intermittent line indicates the lower range of normal lymphocyte count.

The laboratory tests showed normal white cell counts, except for marked lymphopenia (Figure 1), reduction of CD4+ cells (ranging from 50 to 300 cells/μL), increase of fetal hemoglobin, (6.4–13.9% during follow-up), supposedly related to reticulocytosis. While autoantibodies, including Anti-Nuclear Antibodies, Extractable Nuclear Antigen, Anti-DNA antibodies, Anti-Smooth Muscle Antibodies, Anti-phospholipid Antibodies, complement factors, and serum immunoglobulins were within the normal ranges. Immunological screening for celiac and thyroid disease were also negative. Serologic tests for Parvovirus B19, EBV, CMV, and Waaler-Rose test, were consistent with prior infection or non-immunized state. The fecal occult blood test was negative; chest radiograph, echocardiography and abdomen ultrasound were all unremarkable.

She underwent also bone marrow aspirate, that showed normal proportion of myeloid cells, presence of megakaryocytes, and mild dyserythropoiesis, without evidence of cytogenetic abnormalities.

During corticosteroid therapy, hemoglobin levels slowly increased and Coombs test became negative within 3 months. Prednisone (1 mg/kg/bid) was continued for 10 months with progressive tapering. However, this therapy was associated with multiple side effects, such as alopecia, weight gain, moon face, stretch marks, including lymphopenia. This was also associated with reduction of IgG concentrations (IgG 520, n.v. 640–1909 mg/dl; IgM 115, n.v. 59–297 mg/dl, IgA 121, n.v. 61–301 mg/dl) which prompted us to start IVIG every 4 weeks for 3 months. In addition, because of the risks related to lymphopenia and to prolonged immunosuppressive therapy with corticosteroids, prophylaxis with cotrimoxazole and acyclovir was prescribed.

In order to exclude an underling immunodeficiency, we examined lymphocyte subsets. Flow cytometric immunophenotyping showed marked alterations in the distributions of the T-lymphocytes (Table 1): reduction of the CD3+CD4+ T cells (19.7%), especially the Recent Thymus Emigrants (RTE) CD4+ T Cells (2.9%), and increase of the TCR γ/δ T cells (24.7%). Transitional B cells (0.4%), and terminal differentiated B cell subsets (0.06%) were reduced, while Switched Memory B cells (34.2%) were increased. T-cell Receptor Excision Circles (TRECs) and Kappa-deleting Recombination Excision Circles (KRECs) were below normal range, while analysis of the T-cell receptor repertoire showed a marked monoclonal expansion of TCRBV6 expressing cells. Increase of TCR γ/δ T cells could be related to many conditions, including infections. CD3^+^/CD4^−^/CD8^−^ (double negative T cells) subset were in the normal range, thus ruling out the hypothesis of Autoimmune lymphoproliferative syndrome.

**Table 1:** Lymphocyte subsets during corticosteroid therapy^*^.

**T-cell Subsets**	**%**	**Normal range (10–16 years-old)**
T-Lymphocytes CD3+	76.2	60.5–79.8
CD3+CD4+	**19.7**	30.3–48.3
HLADR+	4.8	1.4–17.6
Naive CD45RA+CCR7+	28.9	34.3–74.6
RTE CD45RA+CCR7+CD31+	**2.9**	21.1–63.5
Central Memory CD45-CCR7+	34.7	13.0–43.5
Effectory Memory CD45RA-CCR7-	31.2	8.5–28.1
Terminal Differentiated CD45RA+CCR7-	5.2	0.7–6.6
CD3+CD8+	30.7	13.8–37.5
HLADR+	11.8	2.1–52.0
Naive CD45RA+CCR7+	32.7	26.7–72.9
Central Memory CD45-CCR7+	2.0	1.2–11.6
Effectory Memory CD45RA-CCR7-	15.8	6.0–53.6
Terminal Differentiated CD45RA+CCR7-	49.6	3.9–72.0
CD4+CD8+	0.3	0.1–3.1
CD4-CD8-	17.4	1.9–25.8
TCR γ/δ	**24.7**	0.5–21.5
B-Lymphocytes CD19+	21.1	5.7–19.7
RBE CD38^hi^CD21^dim/lo^CD10+	**0.4**	15.0–35.3
Naive CD38^dim/lo^CD21^hi^CD10-CD27-	36.4	33.8–79.6
CD19^hi^CD21^lo^	5.9	1.1–10.0
Switched Memory IgD-CD27+	**34.2**	2.7–20.6
IgM Memory IgD+CD27+	18.9	3.5–24.1
Terminal Differentiated CD38^hi^CD27^hi^CD21^lo^	**0.06**	0.16–8.7
PC CD38^hi^CD20-CD138+	0.06	0.04–3.2
NK-cells CD3- CD16+CD56+	**1.9**	4.6–27.8

* *(October 2014: total lymphocytes 620 cell/μl)*.

Next, we analyzed by next generation sequencing (Ion Torrent pgm, Thermo Fischer) a panel of genes that are associated with SCID or CID/lymphopenia. Genetic analysis revealed an heterozygous mutation in exon 10 of the gene encoding for adenosine deaminase (*ADA*, p.S291L c.561C>T), while other genes that are associated with autoimmune diseases, including LRBA and CTLA4 were negative for mutations. Sanger sequencing of ADA gene coding and non-coding flanking regions revealed the same mutation. The assessment of adenosine and deoxyadenosine nucleotides levels in the patient at the time of lymphopenia revealed a moderate increase of both metabolites: adenosine was 3.6 μMol/ml RBC (n.v. 0.8–1.6) while deoxyadenosine was 0.006 uMol/ml RBC (n.v. < 0.005), respectively. Total AXP and dAXP in RBC were quantified by high performance liquid chromatography measuring the levels of Ado and dAdo formed by treating neutralized acid extracts with alkaline phosphatase and venom phosphodiesterase (18).

Analysis of ADA activity in red blood cells lysates, as measured by spectrophotometric analysis of the absorbance change at 265 nm (19), was reduced but within the normal range (0.8 U/g Hb; n.v. 0.8–2.5) while PNP activity in red blood cells lysates was normal (200 μ/g Hb; n.v >330). Analysis of ADA activity, of total AXP and dAXP in RBC in her parents were normal; unfortunately, their genetic status has not been investigated for parental decision.

After corticosteroid tapering the patient's total numbers of lymphocytes gradually increased and reached normal levels (Figure 1). One year after stopping corticosteroid therapy lymphocyte count was 1.410 cells/μL, with 550 CD4+cells/uL. Thus, she interrupted the prophylactic therapy with acyclovir and cotrimoxazole.

## Discussion

We report the case of a 14-year-old girl with acute onset autoimmune hemolytic anemia, associated with marked but transient lymphopenia and a heterozygous mutation in *ADA* gene (p.S291L).

After detailed investigations we found adenosine and deoxyadenosine nucleotides levels slightly above the threshold and border-line ADA activity in red blood cells lysates while the patient was receiving the corticosteroid therapy, whereas ADA metabolites were in the normal range in her parents. The same mutation identified in this patient (S291L) in a heterozygous state was previously reported in a SCID patient with compound heterozygosity of ADA gene (20, 21), suggesting that this genetic variation is considered to be pathogenic of ADA deficiency. We hypothesize that a mono-allelic ADA mutation might be a predisposing factor of lymphopenia. In particular, it is likely that during the treatment with corticosteroid, this specific heterozygous variation of ADA gene might result in conformational change of the catalytic site of ADA, and to rapid degradation of the enzyme thereby leading to increase of toxic metabolites. However, further studies in carriers of ADA mutations will be needed to support this hypothesis.

Many authors reported atypical clinical onset of ADA deficiency, sometimes presenting with autoimmune manifestations (22). They might be the result of lymphopenia, alteration of the structure and functions of the thymic microenvironment, defects in cell death and the altered purine metabolism interfering with normal regulatory T-cell function (17). Notarangelo et al. (16) described a case of a 2.3-year-old girl with complete lack of ADA activity who presented with severe atopic dermatitis and insulin-dependent diabetes mellitus, but only mild recurrent infections. A child with SCID secondary to ADA deficiency, who presented with severe hypothyroidism during early infancy was described by Nagpala et al. (23).

Nomura et al. (24) reported a case of neutropenia and myeloid dysplasia in a patient with delayed-onset ADA deficiency and a previous diagnose of acute disseminated encephalomyelitis (25).

ADA deficiency was diagnosed by Nikolajeva et al. (4) in four patients, whose presenting feature was Hemolytic Uremic Syndrome (HUS). The hypothesis is that infections, dysregulations of complement function, autoimmunity and impaired metabolism of children with ADA-SCID could contribute to the development of HUS.

Two cases of ADA deficiency in adults were reported by Ozsahin et al. (15). One patient was a 39-year-old woman who had frequent infections, lymphopenia, and recurrent hepatitis as a child, then developed chronic sinopulmonary infections, including tuberculosis, and hepatobiliary disease; she died of viral leukoencephalopathy at 40 years of age. The second patient was a healthy 28-year-old man with normal immune function, who was identified after his niece died of SCID. Both patients were heteroallelic for missense ADA mutations.

These studies suggest that ADA deficiency could be a predisposing factor to autoimmunity and that the clinical presentation is often variable, delayed, and atypical.

Our case is the first reported case of ADA heterozygous mutation in a patient presenting with autoimmune manifestation and severe lymphopenia following corticosteroid treatment. The increased levels of toxic metabolites and the ADA activity at the lower range of normality suggest a possible role of the pathogenic variant in the clinical phenotype presented by the patient, characterized by autoimmunity and development of deep lymphopenia during steroidal treatment, in the absence of recurrent infections. Nevertheless, the genetic characterization of the patient's pedigree remains incomplete for the unavailability to perform genetic analysis for familiar decision. Our case report suggests that genetic analysis of ADA gene, and analysis of ADA metabolites and ADA activity might be useful in patients who develop a phenotype very suggestive of primary immunodeficiency, as deep lymphopenia, during steroidal treatment for autoimmune manifestations. After performing an accurate genetic analysis and biological validation of heterozygosity, the study of future cases might help to define the role of ADA enzymatic pathway in the pathogenesis of autoimmunity and to elucidate the whole spectrum of ADA deficiency.

## Ethics statement

This study was approved by the Ethical Committee at the ASST Spedali civili, Brescia.

## Author contributions

ST took part to the immunological assessment of the patient, and wrote the article. PC diagnosed the patient with acute haemolytic anemia, admitted her to the hospital, and started the specific therapy. She performed an extensive workup in order to identify a possible cause of the disease and she planned the patient's follow-up during and after the corticosteroid therapy. SG performed for the patient of the next generation sequencing of a panel of genes associated with SCID or CID/lymphopenia. AL assessed the adenosine and deoxyadenosine nucleotides blood levels and analyzed the ADA and PNP activity in red blood cells lysates in the patient and her parents. AB diagnosed the patient with acute haemolytic anemia, admitted her to the hospital and started the specific therapy, and supervised the study. RB planned the immunological investigations to rule out the causes of the severe lymphopenia the patient developed during corticosteroid therapy and supervised the study.

### Conflict of interest statement

The authors declare that the research was conducted in the absence of any commercial or financial relationships that could be construed as a potential conflict of interest.
